# Concussion leads to opposing sensorimotor effects of habituation deficit and fatigue in zebrafish larvae

**DOI:** 10.1093/braincomms/fcae407

**Published:** 2024-11-13

**Authors:** Laura Köcher, Carolina Beppi, Marco Penner, Samuel Meyer, Stefan Yu Bögli, Dominik Straumann

**Affiliations:** Neuroscience Center Zurich, University of Zurich and ETH Zurich, 8057 Zurich, Switzerland; Department of Neurology, University Hospital Zurich and University of Zurich, 8091 Zurich, Switzerland; Clinical Neuroscience Center, University Hospital Zurich and University of Zurich, 8091 Zurich, Switzerland; Neuroscience Center Zurich, University of Zurich and ETH Zurich, 8057 Zurich, Switzerland; Department of Neurology, University Hospital Zurich and University of Zurich, 8091 Zurich, Switzerland; Clinical Neuroscience Center, University Hospital Zurich and University of Zurich, 8091 Zurich, Switzerland; Department of Neurology, University Hospital Zurich and University of Zurich, 8091 Zurich, Switzerland; Neuroscience Center Zurich, University of Zurich and ETH Zurich, 8057 Zurich, Switzerland; Department of Neurology, University Hospital Zurich and University of Zurich, 8091 Zurich, Switzerland; Clinical Neuroscience Center, University Hospital Zurich and University of Zurich, 8091 Zurich, Switzerland; Department of Neurology, University Hospital Zurich and University of Zurich, 8091 Zurich, Switzerland; Clinical Neuroscience Center, University Hospital Zurich and University of Zurich, 8091 Zurich, Switzerland; Neuroscience Center Zurich, University of Zurich and ETH Zurich, 8057 Zurich, Switzerland; Department of Neurology, University Hospital Zurich and University of Zurich, 8091 Zurich, Switzerland; Clinical Neuroscience Center, University Hospital Zurich and University of Zurich, 8091 Zurich, Switzerland

**Keywords:** zebrafish larvae, concussion, traumatic brain injury, habituation, behaviour

## Abstract

Concussion, or mild traumatic brain injury, is caused by sudden mechanical forces impacting the brain either directly or through inertial loading. This can lead to physical, behavioural and cognitive impairments. Despite concussion being a significant health issue, our understanding of the relationship between initial impact force and the subsequent neurological consequences is not well understood. Previously, we established a model of concussion in zebrafish larvae. Here, we further investigate concussions of varying severities in zebrafish larvae using linear deceleration. Using an acoustic assay to monitor the larval sensorimotor behaviour, we found that different parameters of the resulting escape behaviour are modulated by the impact force of the preceding concussive insult. To investigate the relative contributions of habituation performance and fatigue on the escape response behaviour, we constructed a neurocomputational model. Our findings suggest that a concussive impact initially affects habituation performance at first and, as the impact force increases, fatigue is induced. Fatigue then alters the escape response behaviour in an opposing manner.

## Teaser

Köcher *et al*. conducted a study on zebrafish larvae to model concussions of varying severities. Their findings revealed that the escape behaviour parameters were influenced by the force of the concussive impact. Specifically, they observed that a concussive impact impairs habituation performance and that higher impact forces lead to additional fatigue in the larvae.

## Graphical Abstract

**Graphical Abstract:**
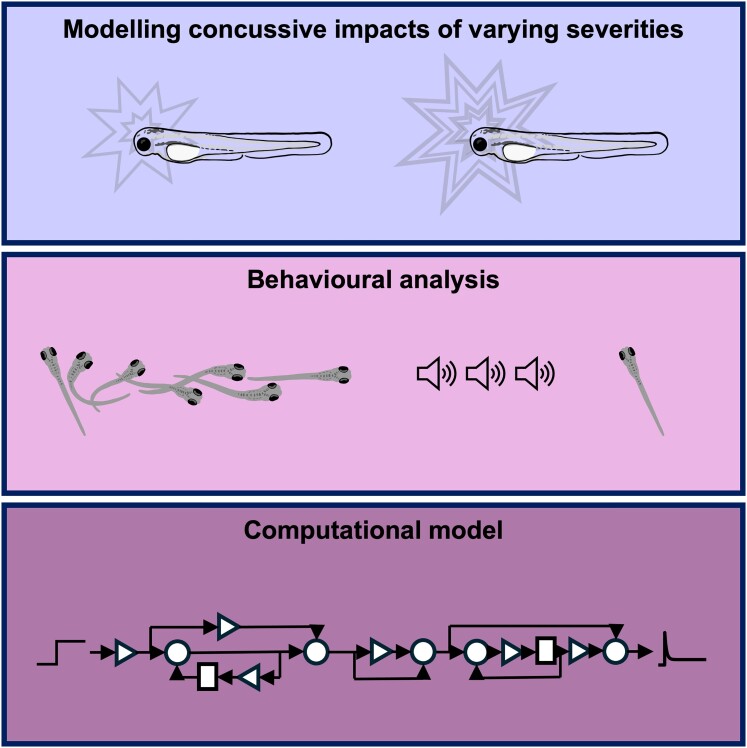

## Introduction

For an animal, filtering relevant from inconsequential information is crucial for enhancing its selective attention to noteworthy environmental features. This sensory filtering demands rapid processing of incoming information and engages various neural mechanisms at multiple levels within the nervous system.^1^ Habituation, the ability to suppress responses to repetitive non-salient stimuli resulting in a progressive decline of the response frequency and/or magnitude, allows an organism to conserve energy while remaining attentive. In many cases, this decline is exponential.^2^ Habituation is the simplest form of non-associative learning and is evolutionarily conserved across vertebrates.^3^ An established paradigm of habituation is the suppression of the acoustic startle response, which is well documented in zebrafish.^4-6^ In zebrafish larvae, an acoustic stimulus triggers a defensive escape manoeuvre. This escape manoeuvre consists of a unilateral contraction of the trunk muscles contralateral to the stimuli, initiated by the Mauthner cells (M-cells)^7,8^ and is followed by a fast forward movement away from the threat, mediated by bilateral activation of the nucleus of medial longitudinal fascicle.^9^ This escape response in larval zebrafish exhibits habituation.^10^ We previously described habituation of the escape response in larval zebrafish as a single-exponential decay.^11^ Deficient filtering of anticipated stimuli is common in many neurological disorders.^12-16^ Distinct from other neurological disease, traumatic brain injury is caused by a mechanical insult to the head—such as due to traffic accidents, falls or sport injuries—that applies extreme forces to the brain.^17^ Traumatic brain injuries are associated with cognitive impairments, including deficits in selective attention.^18^ These impairments have been observed in a dose-dependent manner, correlating with the severity of the injury.^19-21^ However, there is limited understanding of the relationship between the physical properties of an insult and the extent of the ensuing neurological dysfunction. Furthermore, the mechanisms underlying these dysfunctions are largely unknown. Animal models allow to investigate these processes in greater detail. Recently, it has been shown that habituation of the acoustic escape response in larval zebrafish is impaired after a concussive impact.^22^ However, it remains elusive whether habituation progressively reduces with increasing severity of the traumatic brain injury given by the physical properties of an insult. Here, we investigated the relationship between low- and high-impact insults and neurological deficits of the acoustic escape response habituation in zebrafish larvae using a biomechanical-induced model of concussion.

## Materials and methods

### Zebrafish maintenance

All experiments involving zebrafish (*Danio rerio*) were conducted in accordance with the animal welfare guidelines set by the Federal Veterinary Office of Canton Zurich (ZH190/2020, 32971). Adult zebrafish were reared and maintained under standard housing conditions under a 14-h light/10-h dark cycle at 27°C. Embryos were generated from natural mattings. For all experiments, zebrafish larvae of the Wild India Kolkata strain were used. Embryos were raised in E3 medium^23^ at 28°C on a 14-h light/10-h dark cycle. For each experiment, embryos were collected from several mating pairs, and clutch populations were evenly mixed between injury and control groups during experiments.

### Experimental apparatus

The apparatus consisted of a linear motor device (S01–72/500, LinMot, Catalog# 0150-1874) with a stator (PS01-23 × 160H-HP-R, LinMot, Catalog# 0150-1254) mounted on an impact-absorbing stone table and a vertical moving slider (diameter: 12 mm; length: 480 mm, PL01-12 × 480/440-HP, LinMot, Catalog# 0150-1524). A customized 3D-printed cylindrical holder was fixed on the top of the moving component to hold the E3 medium filled with capsule, containing the larvae, during the movement executions. The motion trajectories were programmed in MATLAB (Version R2023b, MathWorks) as input for the linear motor to induce the movement executions. The motor device was paired and controlled with an inbuilt LinMot-Talk software (Version 6.9, LinMot), which controlled the linear motor with the input expressed in change in position (mm) over time (sampling rate: 0.001). To ensure the reliability of the movement executions, a built-in digital oscilloscope was used as readout.

Two different motion trajectories, Trajectory 1 and Trajectory 2, to induce a low- or high-impact injury, respectively, were used for the experiments with following features, respectively: 129.67 and 130.03 mm length, ∼134 and ∼122 ms duration, 2.58 and 3.06 m/s peak velocity, 914.30 and 1645.60 m/s^2^ peak acceleration and 594 799.99 and 1 414 000.00 m/s^3^ peak jerk.

### Concussive impact

At 5 days post-fertilization, zebrafish larvae were randomly assigned into injury group (*n* = 24) and control (*n* = 24) group. After the pre-injury baseline behavioural testing, larvae of both the injury and control groups were transferred into two separate identical transparent cylindrical polystyrene capsules (48 × 52 mm, 60 ml, Semadeni) filled with E3 medium and closed by excluding air bubbles. The capsule containing the larvae of the injury group larvae was placed and fixed on top of the moving component of the experimental apparatus. Upon activation, the desired motion trajectories were executed by the linear motor. At the same time, the control larvae were kept inside the capsule without any movement execution of the capsule. Immediately after execution of the motion trajectories, larvae of the injury and control groups were transferred into individual wells of the same 48-well plate. Individual larvae displaying any physical damage (gross morphological changes and loss of body parts) were excluded from further experiments and immediately euthanized using tricaine methanesulphonate (Sigma-Aldrich, Catalog# E10521).

### Behavioural experiments

Behavioural experiments were performed at six different timepoints: pre-injury (referred as pre-injury baseline), 5 min, 1 h, 2, 24 and 48 h post-injury. Individual larvae of the injury and control groups were randomly distributed into separate wells of the same 48-well plate. Wells were filled with E3 medium. Larvae were allowed to acclimatize for 10 min (exception of the 5 min post-injury timepoint) within the ZebraBox recording chamber (ViewPoint) before start of the behavioural assay. The recording chamber was placed in a separately isolated room to ensure that background noise was reduced to a minimum. Temperature within the isolated room was kept around 26°C. In-between the different recording timepoints larvae were placed into an incubator at 28°C.

Larvae of the control and injury groups were simultaneously recorded. During the recording, the recording chamber was kept in darkness but was illuminated with infra-red light. Movements were tracked in bins of 1 s by tracking three different speed categories (2, 2–20 and >20 mm/s) using the zebrafish tracking software ZebraLab (ViewPoint). To distinguish between swim bouts and acoustic stimuli–induced escape manoeuvres, escape responses were defined as movements above 20 mm/s during an acoustic/vibratory stimulus. Velocity of escape movements was therefore empirically estimated in advance by comparing video data at 1000 fps of the larval acoustic startle responses with the tracking output of the software.

Acoustic/vibratory stimuli were elicited using a behavioural platform connected to an amplifier (CS-PA1 MK, Dynavox). The behavioural assay, adapted from Wolman *et al*.^3^ and slightly modified, consisted of 3 phases with a total of 50 identical acoustic vibratory stimuli. The first phase consisted of 10 stimuli at a sub-threshold low-level intensity (300 Hz, 20 dB) spaced at 60 s inter-stimuli interval (ISI) to assess acoustic response sensitivity. The second phase, the ‘pre-habituation phase’, consisted of 10 stimuli (300 Hz, 108 dB) with 60 s ISI to determine baseline responsiveness to the acoustic stimuli. The last phase, the ‘habituation’ phase, consisted of 30 stimuli (300 Hz, 108 dB) spaced at 1 s ISI to evoke short-term habituation. Each phase was separated by a 5-min break during which spontaneous activity was recorded. The acoustic stimuli were of 500 ms duration with 300 Hz waveforms. Intensities of the acoustic stimuli were empirically identified to either elicited escape responses in ∼20% of the time in control larvae for sub-threshold intensities or >80% escape responses in above threshold intensities. Individual larvae that responded to <40% of the stimuli during the pre-habituation phase with an escape response were classed as non-responders and excluded from further analysis. There was no significant difference in number of non-responders between the three groups.

Behavioural experiments were replicated three times on different days at the same time of the day. Experiments for the two different injury groups, subjected to motion Trajectory 1 or 2, were performed separately, each including a control group. Results of the triplicates were added together (control, *n* = 136; low-impact injury, *n* = 70 and high-impact injury, *n* = 69).

### Quantitative modelling of the behavioural tracking

For each individual larvae, the distance of the escape response over each stimulus of the habituation phase was fitted with a single-exponential decay curve $\left[f(x)=a\times e^{-bx}+c\right]$ in MATLAB using lsqcurvefit function for non-linear curve fitting in least-squares manner. Habituation learning was modelled using a single-exponential decay curve, based on prior studies.^24-27^ From each fitted curve, three parameters were extracted: amplitude, decay constant and offset. Amplitude was defined as the distance of the escape response during the first habituating stimulus. Decay constant corresponds to the number of stimuli required for the amplitude to fall to 1/*e* of its value. Decay constant correlates to the speed of habituation, i.e. the less number of stimuli required for an individual to suppress a succeeding response, the greater the ability of non-associative learning. The offset accounts for the vertical shift of the curve. To verify that the data from both, control and injured larvae, can be best explained by a single-exponential decay model, the goodness of fit was evaluated across various fitting models. Metrics such as Akaike information criterion, corrected Akaike information criterion, consistent Akaike information criterion, Bayesian information criterion, Loglikelihood and adjusted *R*^2^ were compared among linear, single-exponential without *a*, *b* and/or *c* and double-exponential fits of the data. Additionally, it was verified that the data from both groups, control and injured, exhibited similar performance in terms of curve fitting.

Acoustic baseline responsiveness and acoustic sensitivity were calculated for each individual larvae as area under the curve for the distance of the escape response over the 10 pre-habituation stimuli or 10 sub-threshold low-level intensity stimuli, respectively. Spontaneous activity was calculated for each individual larvae as distance moved over a total of 10 min, including movements from all speed categories, during the 5-min break between the first and second phase and the second and third phase of the behavioural assay.

### Neurocomputational model of the escape response behaviour

The neurocomputational model was simulated in Simulink (Version R2023b, MathWorks) and consisted of two sequential processes: a process that implemented habituation and a process that mimicked the effect of fatigue. A step signal, representing the acoustic/vibratory stimuli (0.5 s duration, 1 s ISI), was given as an input for the two processes.

The habituation process was described by three gains: $h_{1}$, $h_{2}$ and $h_{3}$. For simplicity, the entire signal, $h_{1}=1$, was feed to the process. A fraction of the signal, $h_{3}=0.2$, resisting habituation thus represented the offset. A negative feedback loop realized the actual habituation, whereby the gain in the feedback $h_{2}$ determines the decay constant $1/h_{2}$. For cases of impaired habituation, $h_{2}$ decreases.

The fatigue process was described by three gains: $f_{1}$, $f_{2}$ and $f_{3}$. In absence of fatigue, the entire signal transits the process unchanged with $f_{1}=0$, $f_{2}=0$ and $f_{3}=1$. In presence of fatigue, all three gains change to $f_{1}>0$, $f_{2}>0$ and $f_{3}<1$, and the offset of process amounts to $1-f_{3}$. Increasing fatigue was implemented with another negative feedback loop, whereby the integrator was placed before the feedback branch. Thus, the feedback loop acted as a low-pass filter. The gain for the integrator $f_{2}$ determines the growth constant of the fatigue $1/f_{2}$.

As a result of two sequential exponential processes, habituation and fatigue, the output of the neurocomputational model was characterized by two decay constants, $1/h_{2}$ and $1/f_{2}$, and therefore follows a two-phase exponential decay. However, biological variation did not allow to reliably fit the experimental data with a two-phase exponential decay formula. Therefore, the distance of the escape responses from the behavioural tracking data was fitted into a single-exponential decay curve $f(x)=\;a\times e^{-bx}+c$, as described in the section above.

### Statistical analysis

Statistical analyses of the data extracted from the behavioural tracking and Pearson’s χ^2^ test to test mortality rates were performed using R Studio. Shapiro–Wilk test was used to test whether the data extracted from the behavioural tracking were normally distributed, and Levene’s test was used to test the homogeneity of the variances. If the data were normally distributed, significance was assessed using ANOVA with Dunnett’s test for multiple comparisons. If the data were not normally distributed, Kruskall–Wallis test with Dunn’s multiple comparisons test was used. For all experiments, significance was taken as *P < 0.05, **P < 0.01 and ***P < 0.001. All graphs were plotted in MATLAB.

## Results

### Modelling concussive impacts in zebrafish larvae

**Figure 1:**
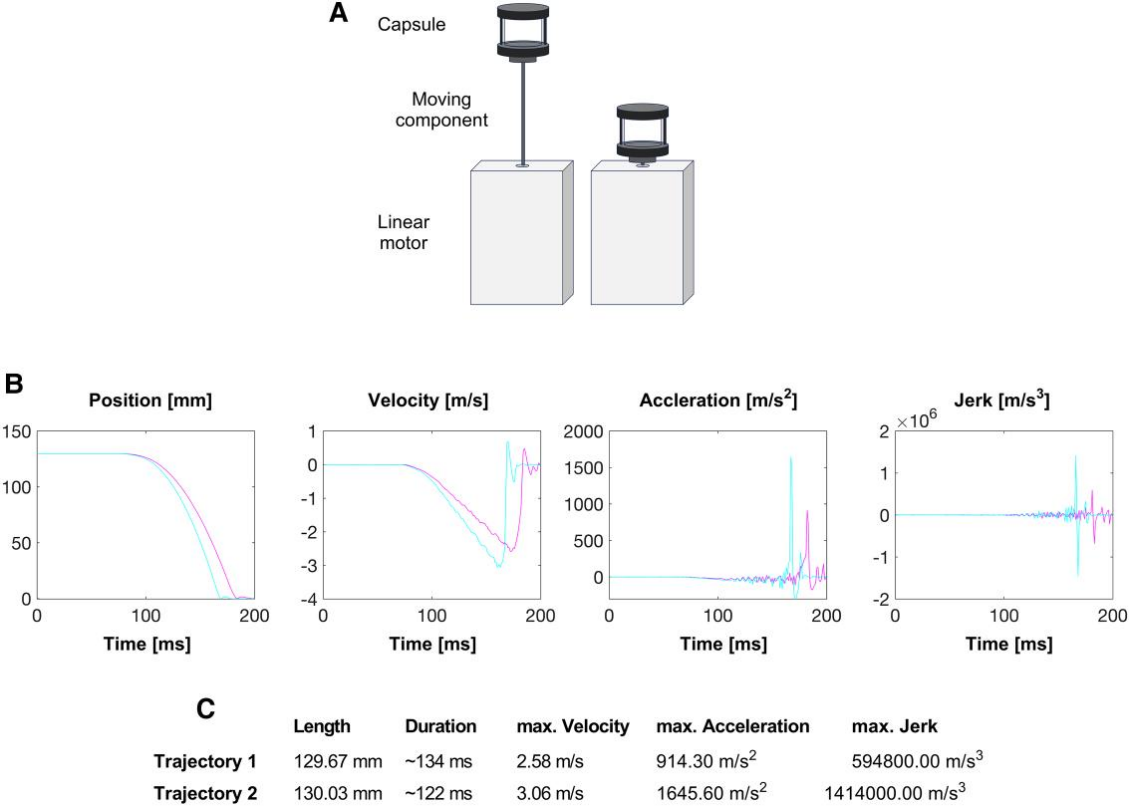
**Modelling concussive impacts in zebrafish larvae.** (**A**) Illustration of the experimental apparatus to induce a low- or high-impact insult in zebrafish larvae. (**B**) Motion Trajectories 1 and 2 were executed by the linear motor and used to induce a low- or high-impact insult, respectively. Both motion trajectories plotted as function of time (ms): position, velocity, acceleration and jerk. (**C**) Features of both motion trajectories: length, duration, maximal velocity, maximal acceleration and maximal jerk.

To characterize the relationship between the ensuing neurological consequences and the preceding mechanical properties of a concussive impact, we subjected zebrafish larvae to linear deceleration to model a concussion. Two different motion trajectories, Trajectory 1 and Trajectory 2, were programmed to induce either a low- or high-impact injury, respectively. The motion trajectories were provided to the linear motor as input to induce the defined movement executions. Larvae were then subjected to a deceleration movement defined by the respective motion trajectory (Fig. 1A and B). The parameters of the two motion trajectories were defined to cause a consistent concussive impact while maximizing the survival rate of the larvae. The low-impact injury did not cause any immediate deaths in larvae, whereas 10.14% of the larvae subjected to the high-impact injury had to be excluded directly afterwards, either due to their lack of responsiveness to tapping or due to distinct signs of death. After 48 h, the survival rate was at 87.14% for the larvae subjected to the low-impact injury and at 62.31% for the high-impact injury group, compared with 97.06% for the control group. Mortality rates did not display significant alteration between control larvae, larvae subjected to a low- and high-impact injury (Pearson’s χ^2^ test, *P* = 0.092). Larvae exhibiting morphological defects or that failed to elicited escape responses were excluded from subsequent analyses.

### Quantifying habituation learning in zebrafish larvae

**Figure 2:**
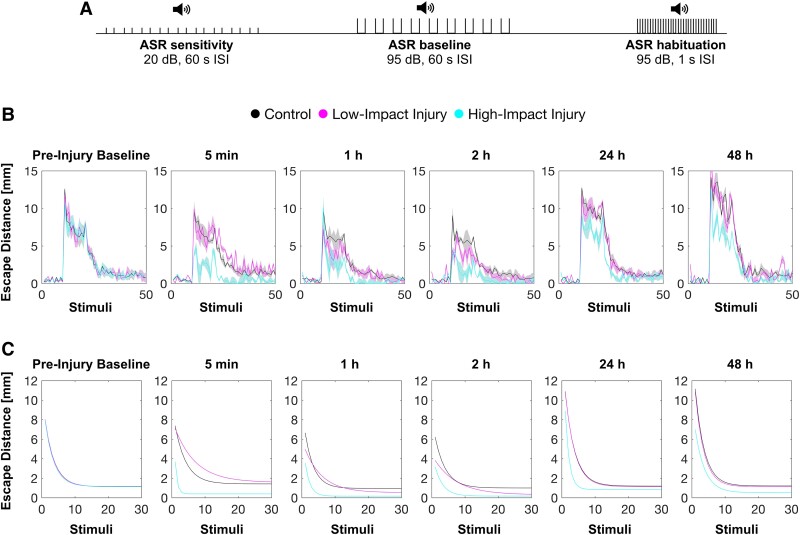
**Longitudinal measurement of acoustic escape response behaviour.** (**A**) Schematic representation of the behavioural assay to measure escape responses upon acoustic stimuli (acoustic startle response). (**B**) Mean escape distance to each stimulus of the behavioural assay for each timepoint and group. (**C**) Fitted single-exponential decay curves of mean escape distance during the habituation phase of the behavioural assay for each timepoint and group: pre-injury (control, *n* = 136; low-impact injury, *n* = 70 and high-impact injury, *n* = 69), 5 min (control, *n* = 136; low-impact injury, *n* = 70 and high-impact injury, *n* = 61), 1 h (control, *n* = 136; low-impact injury, *n* = 70 and high-impact injury, *n* = 60), 2 h (control, *n* = 134; low-impact injury, *n* = 66 and high-impact injury, *n* = 60), 24 h (control, *n* = 132; low-impact injury, *n* = 64 and high-impact injury, *n* = 46) and 48 h after injury (control, *n* = 128; low-impact injury, *n* = 59 and high-impact injury, *n* = 39); data represents mean ± SD.

To investigate the effect of the preceding mechanical insult on the larvae behaviour, which underlies sensory filtering, we adapted an established behavioural assay.^3^ Larvae were exposed to a sequence of 50 acoustic stimuli. This sequence consisted of an initial phase with 10 sub-threshold acoustic stimuli, followed by 10 non-habituating acoustic stimuli at 60 s ISI to determine baseline responsiveness and a final phase of 30 acoustic stimuli spaced at 1 s ISI to evoke short-term habituation (Fig. 2A). We tested the stimuli elicited escape response behaviour before, referred as pre-injury baseline, and after the mechanical insult (Fig. 2B).

The distance of the escape response of each larva during the 30 habituating stimuli of the assay was fitted into a single-exponential decay curve (Fig. 2C). This approach^11,22^ allowed us to describe the habituation process of the elicited escape responses with three parameters: amplitude, decay constant and offset.

**Figure 3:**
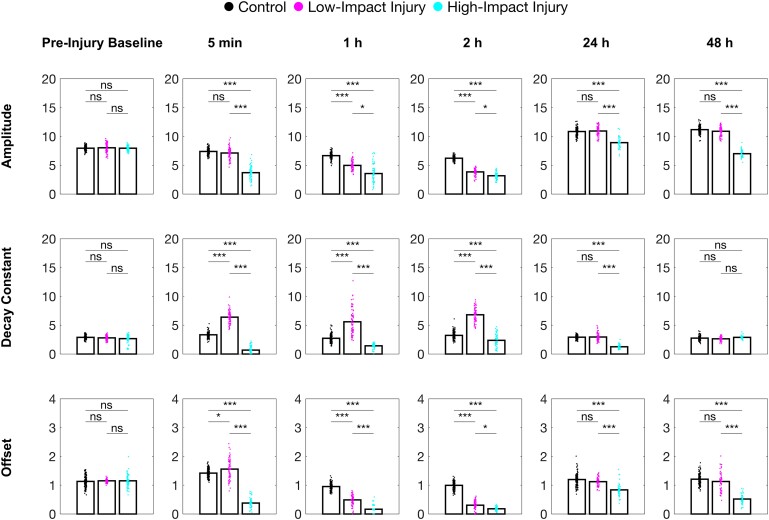
**Quantifying habituation learning in zebrafish larvae.** Extracted parameters of the fitted single-exponential decay curves: amplitude, decay constant and offset. Each dot represents individual larvae, and bar represents mean. ANOVA with Dunnett’s test or Kruskall–Wallis test with Dunn’s multiple comparisons: ns: *P* > 0.05, *P < 0.05, **P < 0.01 and ***P < 0.001. Pre-injury (control, *n* = 136; low-impact injury, *n* = 70 and high-impact injury, *n* = 69), 5 min (control, *n* = 136; low-impact injury, *n* = 70 and high-impact injury, *n* = 61), 1 h (control, *n* = 136; low-impact injury, *n* = 70 and high-impact injury, *n* = 60), 2 h (control, *n* = 134; low-impact injury, *n* = 66 and high-impact injury, *n* = 60), 24 h (control, *n* = 132; low-impact injury, *n* = 64; high-impact injury, *n* = 46) and 48 h after injury (control, *n* = 128; low-impact injury, *n* = 59 and high-impact injury, *n* = 39).

Amplitude is defined as the distance of the escape response upon the first habituating stimulus. Offset represents the steady-state responsivity. Decay constant is defined as the number of stimuli required for the amplitude to fall 1/*e* of its value and correlates to the speed of habituation (Fig. 3). Note that decay constant is considered as being mathematically independent from changes in amplitude or offset, providing that the decay function is approximated to a single exponential.

We found that, immediately after the high-impact injury, larvae displayed a significantly decreased amplitude compared with controls. The low-impact injury, however, did not alter amplitude. Likewise, offset also was significantly decreased subsequently after a high-impact injury. Both amplitude and offset remained significantly lower in larvae subjected to the high-impact injury until the end of the experimental period at 7 days post-fertilization.

Immediately after the low-impact injury, larvae showed slightly increased offset, which then significantly decreased after 1 h and recovered again to comparable levels as in controls after 24 h. Similarly to offset, amplitude of the larval escape response was decreased 1 h after the low-impact injury and returned to similar levels as controls after 24 h.

Interestingly, decay constant developed in opposite directions after low- and high-impact injuries. Subsequently to the low-impact injury, decay constant was significantly prolonged, but after 24 h, decay constant was restored to levels as controls, likewise amplitude and offset. In contrast, following a high-impact injury, larvae displayed significantly shortened decay constant compared with control larvae, but also returned to equal levels in decay constant as control larvae after 48 h.

Together, these results indicate that both amplitude and offset of the escape response decrease with increasing force of the impact. Both, amplitude and offset recover after 24 h, but only after a low-impact injury. Notably, low-impact injuries prolong decay constant of the larvae escape response, while high-impact injuries conversely shorten decay constant.

### Baseline responsiveness is modulated by the force of the concussive impact

**Figure 4:**
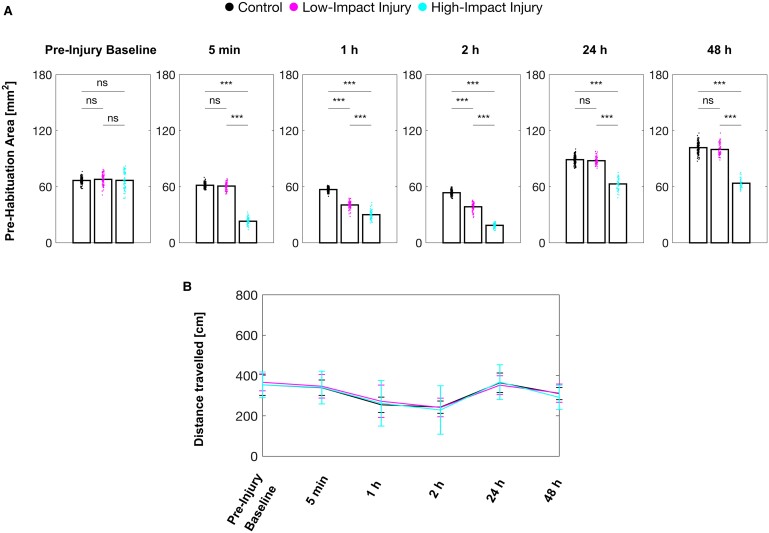
**Baseline responsiveness is modulated by the force of the concussive impact.** (**A**) Acoustic baseline responsiveness measured as area under the curve during the pre-habituation phase of the assay. Each dot represents individual larvae, and bar represents mean. ANOVA with Dunnett’s test for multiple comparisons: ns: *P* > 0.05, *P < 0.05, **P < 0.01 and ***P < 0.001. (**B**) Spontaneous activity in absence of acoustic stimuli, represented as mean ± SD.

To determine the baseline responsiveness to acoustic stimuli, the distance of each larva’s escape response over the 10 non-habituating stimuli was plotted, and the area under the resulting curve was measured (Fig. 4A). Acoustic baseline responsiveness of the larvae subjected to a high-impact injury was significantly reduced 5 min after the injury compared with control larvae, and this reduction persisted until the end of the experimental period, 48 h later. In contrast, larvae subjected to a low-impact injury displayed similar acoustic baseline responsiveness as controls immediately after the injury. However, 1 and 2 h after the low-impact injury, the larval acoustic baseline responsiveness was also reduced but returned to similar levels as controls after 24 h.

We did not observe a significant difference in the larval acoustic response sensitivity for any timepoints after neither a low- nor a high-impact injury. Thus, larval responsiveness towards sub-threshold stimuli was not affected by the mechanical insults we investigated here. Further, acoustic baseline responsiveness was only reduced for an acute period after a low-impact injury, whereas high-impact injuries caused a persistent reduction in acoustic baseline responsiveness.

### Neurological deficiencies do not generalize to other behaviours

Next, we questioned whether other larval zebrafish behaviours also exhibit injury-induced differences. We therefore monitored spontaneous activity in absence of acoustic stimuli (Fig. 4B). The spontaneous activity after low- and high-impact injury was comparable with control larvae at all timepoints. Therefore, the change observed in sensory-evoked motor behaviour caused by a mechanical insult described above does not generalize to other larval behaviour.

### Fatigue contributes to the sensory-evoked motor behavioural outcome

The differential results of decay constant we observed after a low- and high-impact injury, together with persistently reduced baseline responsiveness after the high-impact injury, could be explained by a central or peripheral fatigue. If fatigue and habituation would be affected in parallel, we would not expect fatigue to affect the habituation performance of the escape response behaviour. However, if fatigue and habituation are sequential processes differentially affected by the magnitude of the impact, an inverse correlation between the degree of the fatigue and of the degree of habituation impairment would be expected. To explore the potential contribution of fatigue on the habituation performance, we constructed a simplified neurocomputational model.

**Figure 5:**
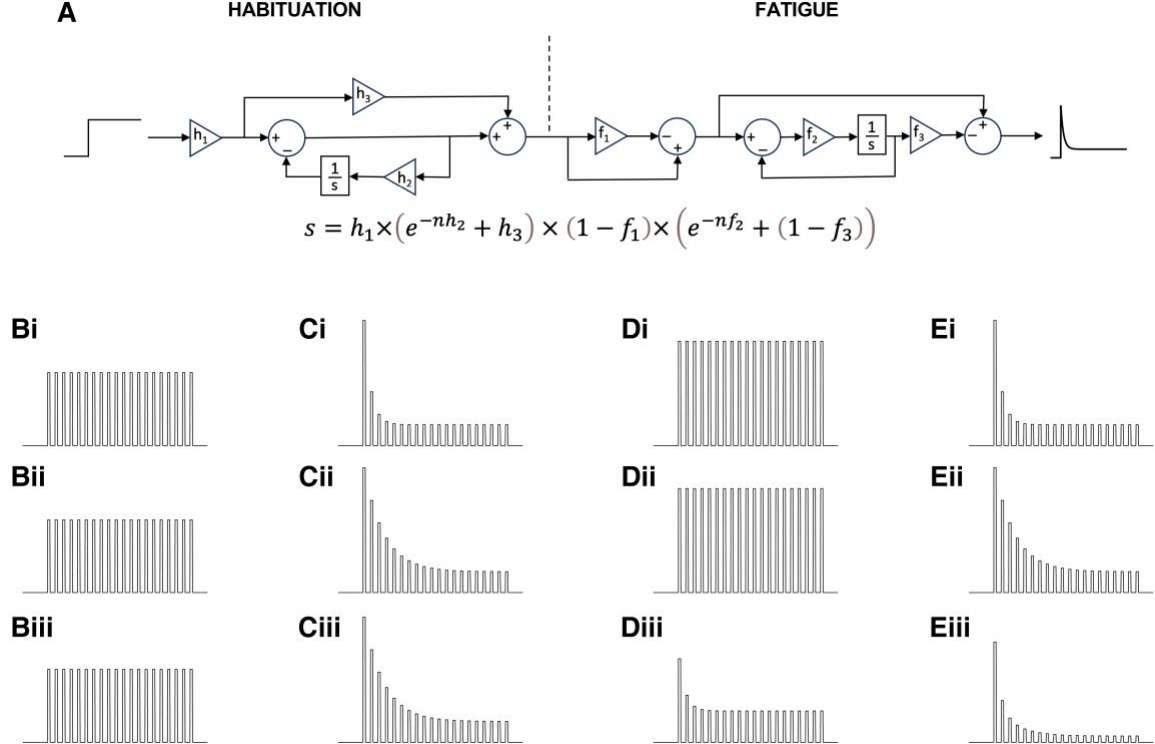
**Neurocomputational model of the escape response behaviour.** Neurocomputational model (**A**) and simulation (**Bi–Eiii**) of the escape response upon repeated acoustic vibratory stimulation. (**A**) The input signal, displayed as frequency step, consists of vibratory stimuli (0.5 s duration, 1 s ISI); for better visibility, the first 20 of the 30 total stimuli are displayed. The signal is first modified by a habituation process consisting of three gains $\left(h_{1},h_{2},h_{3}\right)$ and high-pass filtering by a negative feedback loop with an integrator (1/s), whereby $1/h_{2}$ yields the decay constant of habituation. The resulting signal is routed to a process representing fatigue that is implemented with another three gains $\left(f_{1},f_{2},f_{3}\right)$and low-pass filtering with negative feedback loop after integration (1/s), whereby $1/f_{2}$ yields the constant of fatigue increase. This fatigue signal is then subtracted from the total signal. The final output of the overall model is a high-pass filtered signal with two decay constants and an offset determined by $h_{3}$ and $f_{3}$. The algebraic formula of the model allows computing the distance (*s*) of the escape response following each stimulation (*n*). (**Bi–Biii**) Input signal consisting of 20 acoustic vibratory stimuli. Output signal of the habituation process: (**Ci**) under normal habituation with *h*_1_ = 1, *h*_2_ = 1⁄1.3 and *h*_3_ = 0.2 and (**Cii** and **Ciii**) under impaired habituation with *h*_1_ = 1, *h*_2_ = 1⁄4 and *h*_3_ = 0.2. Output signal of the fatigue process, when bypassing the habituation process: (**Di** and **Dii**) under absence of fatigue with $f_{1}$ = 0, $f_{2}$ = 0 and $f_{3}$ = 1 and (**Diii**) in presence of fatigue with *f*_1_ = 0.5, *f*_2_ = 0.8 and *f*_3_ = 0.4. Final output signal after the habituation and fatigue processes: (**Ei** and **Eii**) output signal remains identical to the output signal coming from the habituation process and (**Eiii**) output signal with shortened decay constant due to the counteracting effect of the fatigue process on the signal coming from the habituation process.

This simple neurocomputational model simulates habituation and fatigue as linked processes connected in series (Fig. 5A). According to the output of the model, corresponding to the predicted escape response behaviour, decay constant shortens as fatigue increases, while amplitude and offset decrease. Hence, under high degrees of habituation and in absence of fatigue, such as expected in healthy larvae not subjected to any mechanical insult, the model predicts a relatively short decay constant (Fig. 5Ei). Impaired habituation translates to prolonged decay constant (Fig. 5Eii). When combining impaired habituation with excessive fatigue, the model predicts shortened decay constant along with lower amplitude and offset (Fig. 5Eiii). Thus, the model was able to explain how varying degrees of fatigue, determined by the impact force, may influence habituation performance of the escape response behaviour.

## Discussion

Traumatic brain injury is one of the leading causes for hospitalization, morbidity and mortality across all ages.^28^ Clinically, traumatic brain injury is classified according to the symptoms into three categories: mild, moderate and severe.^29^ Mild traumatic brain injury, often termed as concussion, accounts for 70–85% of all traumatic brain injuries.^30^ Symptoms caused by concussion may include headache, nausea, dizziness and cognitive, emotional and sleep difficulties.^31^ Fatigue is commonly reported in concussion and many neurologic diseases, including multiple sclerosis, Parkinson’s disease and myasthenia gravis. Fatigue is described as diminished capacity to perform a task and can originate from central and peripheral mechanisms. While peripheral fatigue occurs distal to the neuromuscular junction, central fatigue refers to processes within motoneurons and the CNS.^32^ Several studies could show that fatigue is distinguishable from other disease-related symptoms such as sleepiness, sleep impairment, depression and apathy.^33-35^

Traumatic brain injury causes structural and functional damage due to both, the initial injury and the multifaceted pathological processes, termed as secondary injury, which occur subsequently after the initial insult and may include neuronal excitotoxicity, oxidative damage, mitochondrial dysfunction and neuroinflammation.^36-39^ Together, the initial insult and its evolving secondary injuries provoke ongoing pathophysiological changes, which result in diverse clinical outcomes progressing for a few weeks, sometimes up to many years. Especially, athletes in collision sports and military personnel are at a high risk to be exposed to head impacts throughout their career.^17,40^ Previous literature reported average concussive impacts in adult athletes around 105 g,^41,42^ whereas lowest concussive impacts were at 42 g.^43,44^ Nevertheless, there is insufficient knowledge between the relation of the mechanical insult and the resulting injury outcome in terms of neurological deficits. Determining the mechanical properties of an insult is a key to understand its neurological consequences. This knowledge may improve more adequate prediction of the injury outcome and thus helps to accustom the respective treatment and diagnosis after the injury.

Mammalian models have so far mostly contributed to the knowledge about the neurological consequences of traumatic brain injuries; however, they come with some drawbacks such as expensive maintenance and ethical hurdles.^45-47^ Although most traumatic brain injury models were established in rodents, recent traumatic brain injury models in zebrafish have found comparable cellular responses as seen in mammals.^48-50^ Zebrafish have a well-developed nervous system including many neuroanatomic structures and neurotransmitter pathways in comparison with mammals.^51-53^ Their transparency at larval stages further allows for non-invasive whole-brain imaging.^54^ Despite their great potential, targeted brain lesion methods to model concussion in larval zebrafish can produce some unaccounted side-effects due to the small size of larval brain. During early development, the zebrafish skull takes several weeks to fully form, leaving the brain only partially covered by bone until the late juvenile stages. In larval zebrafish, the cartilage that is later replaced by bone consists of a relatively thin layer of cells.^55^ Methods acting on the animal’s entire brain would more closely resemble concussive incidents. More recently, we introduced a model of concussion in larval zebrafish.^22,56^ Here, we follow up and induced concussive impacts of different severities in larvae zebrafish to investigate the sensorimotor behavioural consequences.

To that end, we monitored spontaneous activity in absence of acoustic stimuli. Neither low-nor high-impact injury altered larvae spontaneous activity, indicating that generalized motor deficits most likely did not occur.

Decay constant, which correlates to the speed of habituation and thus to the degree of habituation learning, was found prolonged after a low-impact injury and shortened after a high-impact injury. However, amplitude and offset were even more decreased after a high-impact injury than after a low-impact injury. Since habituation is defined as decline in behavioural response to repeated stimuli, independent of sensory adaptation or motor fatigue,^2^ we quantified baseline responsiveness towards acoustic stimuli to address this aspect. Acoustic baseline responsiveness was not immediately altered after the low-impact injury and was only reduced for an acute period. Instead, larval acoustic baseline responsiveness was persistently reduced after a high-impact injury, indicating towards the presence of a central or peripheral fatigue. Given the concurrent decreases in amplitude and offset, we hypothesize that the significant shortening of decay constant observed after high-impact injury is more likely a result of fatigue masking the habituation process.

To test this hypothesis, we created a simplified neurocomputational model simulating the resulting escape response of the larvae to acoustic stimuli, incorporating habituation and fatigue as sequential processes. Our model predicts that the more pronounced the fatigue, the lower the resulting amplitude and offset of the escape response. While impaired habituation prolongs decay constant, the model predicts that if the degree of fatigue increases in parallel to the habituation impairment, decay constant of the escape response shortens. The observed negative correlation between the habituation performance and the magnitude of impact force suggests a model where fatigue operates in series to the habituation process and indicates that different degrees of fatigue affect the overall output of the process in an opposing manner.

Plasticity processes, including structural and synaptic plasticity, are frequently involved in nervous system changes induced by brain injuries such as concussive impacts.^57^ Those processes may involve alterations in synaptic strength or neurotransmitter release. Our results indicate that fatigue evoked by a mechanical insult modulates sensorimotor behaviour. This opens the possibility for multiple subsequent detailed investigations on how behavioural plasticity might be modulated by this observed fatigue and shed light on the physical site where such plasticity processes occur. The mechanism the brain uses to accomplish selective filtering of specific stimuli remains largely unknown. Indeed, it has been shown that habituation involves multiple independent processes that each tune individual components of behaviour.^37^ However, it remains mostly elusive how neurological disorders are manifested in sensorimotor behaviour and the underlying mechanisms that modulate these changes.

Recent studies suggest that even sub-concussive impacts can cumulatively lead to neurodegenerative diseases, such as chronic traumatic encephalopathy.^58,59^ Therefore, investigating the correlation between multiple sub-concussive impacts, the time in-between these impacts and the subsequent neurological consequences may provide valuable insights into the chronic outcomes of concussion.

In contrast to larval stages, juvenile and adult zebrafish exhibit a broad range of social behaviours including shoaling and schooling. The preference to approach and stay near conspecifics begins to emerge around 7 days post-fertilization,^60,61^ while shoaling behaviour becomes apparent at ∼15 days post-fertilization.^62^ However, the ability to swim in a coordinated manner, essential for schooling, has so far only been described in adult zebrafish.^63^ Sub-cortical brain regions play a critical role in coordinating social behaviours, and many of these regions are highly conserved across vertebrates.^64,65^ Therefore, studying social behaviours in adult zebrafish after experiencing a concussive impact at larval stages could reveal important functional consequences for these sub-cortical networks. Since zebrafish hold the capacity for continuous neurogenesis until adulthood,^66^ assessing different behaviours at juvenile and adult stages would provide insights into the underlying neural circuits and their potential to recover from concussive impact.

## Conclusion

In summary, a concussive impact may affect the habituation performance, reflected in prolonged decay constant of the escape response behaviour. Our results demonstrate that with increasing impact forces, fatigue arises soon, persists longer and appears more pronounced. Hence, the more severe the impact-induced fatigue, the smaller amplitude and offset of the escape response towards repetitive acoustic stimuli and contrariwise the shorter decay constant. Thus, fatigue alters the emerging escape response behaviour of the habituation performance in an opposing manner.

## Data Availability

No datasets of standardized datatypes were generated for this study. All original codes generated for this study are openly available at https://data.mendeley.com/datasets/7njhhgwtz9/1. Any additional information required to reanalyse the data reported in this paper is available from the corresponding author upon request.

## References

[1] Ramaswami M . Network plasticity in adaptive filtering and behavioral habituation. Neuron. 2014;82(6):1216–1229. 24945768 10.1016/j.neuron.2014.04.035

[2] Rankin CH, Abrams T, Barry RJ, et al Habituation revisited: An updated and revised description of the behavioral characteristics of habituation. Neurobiol Learn Mem. 2009;92(2):135–138. 18854219 10.1016/j.nlm.2008.09.012 PMC2754195

[3] Wolman MA, Jain RA, Liss L, Granato M. Chemical modulation of memory formation in larval zebrafish. Proc Natl Acad Sci U S A. 2011;108(37):15468–15473. 21876167 10.1073/pnas.1107156108 PMC3174630

[4] Marsden KC, Granato M. In vivo Ca(2+) imaging reveals that decreased dendritic excitability drives startle habituation. Cell Rep. 2015;13(9):1733–1740. 26655893 10.1016/j.celrep.2015.10.060 PMC4680997

[5] Santistevan NJ, Nelson JC, Ortiz EA, et al Cacna2d3, a voltage-gated calcium channel subunit, functions in vertebrate habituation learning and the startle sensitivity threshold. PLoS One. 2022;17(7):e0270903. 35834485 10.1371/journal.pone.0270903 PMC9282658

[6] Pantoja C, Hoagland A, Carroll EC, Karalis V, Conner A, Isacoff EY. Neuromodulatory regulation of behavioral individuality in zebrafish. Neuron. 2016;91(3):587–601. 27397519 10.1016/j.neuron.2016.06.016 PMC4976045

[7] Burgess HA, Granato M. Sensorimotor gating in larval zebrafish. J Neurosci. 2007;27(18):4984–4994. 17475807 10.1523/JNEUROSCI.0615-07.2007 PMC6672105

[8] Korn H, Faber DS. The Mauthner cell half a century later: A neurobiological model for decision-making? Neuron. 2005;47(1):13–28. 15996545 10.1016/j.neuron.2005.05.019

[9] Xu L, Guan NN, Huang CX, Hua Y, Song J. A neuronal circuit that generates the temporal motor sequence for the defensive response in zebrafish larvae. Curr Biol. 2021;31(15):3343–3357.e4. 34289386 10.1016/j.cub.2021.06.054

[10] Eaton RC, Farley RD, Kimmel CB, Schabtach E. Functional development in the Mauthner cell system of embryos and larvae of the zebra fish. J Neurobiol. 1977;8(2):151–172. 856948 10.1002/neu.480080207

[11] Beppi C, Straumann D, Bögli SY. A model-based quantification of startle reflex habituation in larval zebrafish. Sci Rep. 2021;11(1):846. 33436805 10.1038/s41598-020-79923-6 PMC7804396

[12] Penders CA, Delwaide PJ. Blink reflex studies in patients with Parkinsonism before and during therapy. J Neurol Neurosurg Psychiatry. 1971;34(6):674–678. 5158781 10.1136/jnnp.34.6.674 PMC1083500

[13] Braff DL, Geyer MA. Sensorimotor gating and schizophrenia. Human and animal model studies. Arch Gen Psychiatry. 1990;47(2):181–188. 2405807 10.1001/archpsyc.1990.01810140081011

[14] Sinha SP, Avcu P, Spiegler KM, et al Startle suppression after mild traumatic brain injury is associated with an increase in pro-inflammatory cytokines, reactive gliosis and neuronal loss in the caudal pontine reticular nucleus. Brain Behav Immun. 2017;61:353–364. 28089558 10.1016/j.bbi.2017.01.006

[15] Hermann B, Salah AB, Perlbarg V, et al Habituation of auditory startle reflex is a new sign of minimally conscious state. Brain. 2020;143(7):2154–2172. 32582938 10.1093/brain/awaa159 PMC7364741

[16] Papesh MA, Elliott JE, Callahan ML, Storzbach D, Lim MM, Gallun FJ. Blast exposure impairs sensory gating: Evidence from measures of acoustic startle and auditory event-related potentials. J Neurotrauma. 2019;36(5):702–712. 30113267 10.1089/neu.2018.5801 PMC6387566

[17] Faul M, Coronado V. Epidemiology of traumatic brain injury. Handb Clin Neurol. 2015;127:3–13. 25702206 10.1016/B978-0-444-52892-6.00001-5

[18] Dockree PM, Bellgrove MA, ‘Keeffe O, et al Sustained attention in traumatic brain injury (TBI) and healthy controls: Enhanced sensitivity with dual-task load. Exp Brain Res. 2006;168(1-2):218–229. 16044297 10.1007/s00221-005-0079-x

[19] Dikmen S, Machamer J, Temkin N. Mild traumatic brain injury: Longitudinal study of cognition, functional status, and post-traumatic symptoms. J Neurotrauma. 2017;34(8):1524–1530. 27785968 10.1089/neu.2016.4618 PMC5397200

[20] Wilson L, Horton L, Kunzmann K, et al CENTER-TBI participants and investigators. Understanding the relationship between cognitive performance and function in daily life after traumatic brain injury. J Neurol Neurosurg Psychiatry. 2020;92(4):407–417. jnnp-2020-324492. 10.1136/jnnp-2020-324492 33268472

[21] Mollayeva T, Mollayeva S, Pacheco N, D’Souza A, Colantonio A. The course and prognostic factors of cognitive outcomes after traumatic brain injury: A systematic review and meta-analysis. Neurosci Biobehav Rev. 2019;99:198–250. 30641116 10.1016/j.neubiorev.2019.01.011

[22] Beppi C, Penner M, Straumann D, Bögli SY. A non-invasive biomechanical model of mild TBI in larval zebrafish. PLoS One. 2022;17(5):e0268901. 35622781 10.1371/journal.pone.0268901 PMC9140253

[23] Westerfield M . The zebrafish book. A guide for the laboratory use of zebrafish (*Danio rerio*). The zebrafish book. University of Oregon Press; 2000.

[24] Randlett O, Haesemeyer M, Forkin G, et al Distributed plasticity drives visual habituation learning in larval zebrafish. Curr Biol. 2019;29(8):1337–1345.e4. 30955936 10.1016/j.cub.2019.02.039 PMC6545104

[25] Bátora D, Zsigmond Á, Lőrincz IZ, Szegvári G, Varga M, Málnási-Csizmadia A. Subcellular dissection of a simple neural circuit: Functional domains of the Mauthner-cell during habituation. Front Neural Circuits. 2021;15:648487. 33828462 10.3389/fncir.2021.648487 PMC8019725

[26] Fotowat H, Engert F. Neural circuits underlying habituation of visually evoked escape behaviors in larval zebrafish. Elife. 2023;12:e82916. 36916795 10.7554/eLife.82916 PMC10014075

[27] Mancienne T, Marquez-Legorreta E, Wilde M, et al Contributions of luminance and motion to visual escape and habituation in larval zebrafish. Front Neural Circuits. 2021;15:748535. 34744637 10.3389/fncir.2021.748535 PMC8568047

[28] Taylor CA, Bell JM, Breiding MJ, Xu L. Traumatic brain injury-related emergency department visits, hospitalizations, and deaths - United States, 2007 and 2013. MMWR Surveill Summ. 2017;66(9):1–16. 10.15585/mmwr.ss6609a1 PMC5829835 28301451

[29] Teasdale G, Jennett B. Assessment of coma and impaired consciousness. A practical scale. Lancet. 1974;2(7872):81–84. 4136544 10.1016/s0140-6736(74)91639-0

[30] Dewan MC, Rattani A, Gupta S, et al Estimating the global incidence of traumatic brain injury. J Neurosurg. 2018;130(4):1080–1097. 29701556 10.3171/2017.10.JNS17352

[31] Dixon KJ . Pathophysiology of traumatic brain injury. Phys Med Rehabil Clin N Am. 2017;28(2):215–225. 28390509 10.1016/j.pmr.2016.12.001

[32] Kluger BM, Krupp LB, Enoka RM. Fatigue and fatigability in neurologic illnesses: Proposal for a unified taxonomy. Neurology. 2013;80(4):409–416. 23339207 10.1212/WNL.0b013e31827f07be PMC3589241

[33] Barone P, Antonini A, Colosimo C, et al The PRIAMO study: A multicenter assessment of nonmotor symptoms and their impact on quality of life in Parkinson’s disease. Mov Disord. 2009;24(11):1641–1649. 19514014 10.1002/mds.22643

[34] Havlikova E, van Dijk JP, Rosenberger J, et al Fatigue in Parkinson’s disease is not related to excessive sleepiness or quality of sleep. J Neurol Sci. 2008;270(1-2):107–113. 18371981 10.1016/j.jns.2008.02.013

[35] Alves G, Wentzel-Larsen T, Larsen JP. Is fatigue an independent and persistent symptom in patients with Parkinson disease? Neurology. 2004;63(10):1908–1911. 15557510 10.1212/01.wnl.0000144277.06917.cc

[36] Chamoun R, Suki D, Gopinath SP, Goodman JC, Robertson C. Role of extracellular glutamate measured by cerebral microdialysis in severe traumatic brain injury. J Neurosurg. 2010;113(3):564–570. 20113156 10.3171/2009.12.JNS09689 PMC3464461

[37] Frugier T, Morganti-Kossmann MC, O’Reilly D, McLean CA. In situ detection of inflammatory mediators in post mortem human brain tissue after traumatic injury. J Neurotrauma. 2010;27(3):497–507. 20030565 10.1089/neu.2009.1120

[38] Hall ED, Detloff MR, Johnson K, Kupina NC. Peroxynitrite-mediated protein nitration and lipid peroxidation in a mouse model of traumatic brain injury. J Neurotrauma. 2004;21(1):9–20. 14987461 10.1089/089771504772695904

[39] Kim S, Han SC, Gallan AJ, Hayes JP. Neurometabolic indicators of mitochondrial dysfunction in repetitive mild traumatic brain injury. Concussion. 2017;2(3):CNC48. 30202587 10.2217/cnc-2017-0013 PMC6128012

[40] Theadom A, Starkey NJ, Dowell T, et al Sports-related brain injury in the general population: An epidemiological study. J Sci Med Sport. 2014;17(6):591–596. 24602688 10.1016/j.jsams.2014.02.001

[41] Campolettano ET, Gellner RA, Sproule DW, Begonia MT, Rowson S. Quantifying youth football helmet performance: Assessing linear and rotational head acceleration. Ann Biomed Eng. 2020;48(6):1640–1650. 32266597 10.1007/s10439-020-02505-0 PMC7494015

[42] Pellman EJ, Viano DC, Tucker AM, Casson IR, Waeckerle JF. Concussion in professional football: Reconstruction of game impacts and injuries. Neurosurgery. 2003;53(4):799–812. 14519212 10.1093/neurosurgery/53.3.799

[43] Rowson S, Duma SM, Stemper BD, et al Correlation of concussion symptom profile with head impact biomechanics: A case for individual-specific injury tolerance. J Neurotrauma. 2018;35(4):681–690. 29132269 10.1089/neu.2017.5169

[44] Rowson S, Duma SM. Development of the STAR evaluation system for football helmets: Integrating player head impact exposure and risk of concussion. Ann Biomed Eng. 2011;39(8):2130–2140. 21553135 10.1007/s10439-011-0322-5

[45] Meconi A, Wortman RC, Wright DK, et al Repeated mild traumatic brain injury can cause acute neurologic impairment without overt structural damage in juvenile rats. PLoS One. 2018;13(5):e0197187. 29738554 10.1371/journal.pone.0197187 PMC5940222

[46] Pham L, Shultz SR, Kim HA, et al Mild closed-head injury in conscious rats causes transient neurobehavioral and glial disturbances: A novel experimental model of concussion. J Neurotrauma. 2019;36(14):2260–2271. 30843474 10.1089/neu.2018.6169

[47] Bodnar CN, Roberts KN, Higgins EK, Bachstetter AD. A systematic review of closed head injury models of mild traumatic brain injury in mice and rats. J Neurotrauma. 2019;36(11):1683–1706. 30661454 10.1089/neu.2018.6127 PMC6555186

[48] Gan D, Wu S, Chen B, Zhang J. Application of the zebrafish traumatic brain injury model in assessing cerebral inflammation. Zebrafish. 2020;17(2):73–82. 31825288 10.1089/zeb.2019.1793

[49] McCutcheon V, Park E, Liu E, et al A novel model of traumatic brain injury in adult zebrafish demonstrates response to injury and treatment comparable with mammalian models. J Neurotrauma. 2017;34(7):1382–1393. 27650063 10.1089/neu.2016.4497

[50] Alyenbaawi H, Kanyo R, Locskai LF, et al Seizures are a druggable mechanistic link between TBI and subsequent tauopathy. Elife. 2021;10:e58744. 33527898 10.7554/eLife.58744 PMC7853719

[51] Panula P, Sallinen V, Sundvik M, et al Modulatory neurotransmitter systems and behavior: Towards zebrafish models of neurodegenerative diseases. Zebrafish. 2006;3(2):235–247. 18248264 10.1089/zeb.2006.3.235

[52] Wullimann MF, Rupp B, Reichert H. Neuroanatomy of the zebrafish brain. Birkhäuser Basel; 1996. 10.1007/BF00195012 8827327

[53] Panula P, Chen YC, Priyadarshini M, et al The comparative neuroanatomy and neurochemistry of zebrafish CNS systems of relevance to human neuropsychiatric diseases. Neurobiol Dis. 2010;40(1):46–57. 20472064 10.1016/j.nbd.2010.05.010

[54] Kettunen P . Calcium imaging in the zebrafish. In: Islam M, ed. Calcium signaling. Advances in experimental medicine and biology. Springer; 2012:1039–1071. 10.1007/978-94-007-2888-2_48 22453983

[55] Mork L, Crump G. Zebrafish craniofacial development: A window into early patterning. Curr Top Dev Biol. 2015;115:235–269. 26589928 10.1016/bs.ctdb.2015.07.001 PMC4758817

[56] Beppi C, Penner M, Straumann D, Bögli SY. Biomechanical induction of mild brain trauma in larval zebrafish: Effects on visual startle reflex habituation. Brain Commun. 2023;5(2):fcad062. 37006333 10.1093/braincomms/fcad062 PMC10065185

[57] Nishibe M, Barbay S, Guggenmos D, Nudo RJ. Reorganization of motor cortex after controlled cortical impact in rats and implications for functional recovery. J Neurotrauma. 2010;27(12):2221–2232. 20873958 10.1089/neu.2010.1456 PMC2996815

[58] Stein TD, Montenigro PH, Alvarez VE, et al Beta-amyloid deposition in chronic traumatic encephalopathy. Acta Neuropathol. 2015;130(1):21–34. 25943889 10.1007/s00401-015-1435-y PMC4529056

[59] McKee AC, Stern RA, Nowinski CJ, et al The spectrum of disease in chronic traumatic encephalopathy. Brain. 2013;136(Pt 1):43–64. 23208308 10.1093/brain/aws307 PMC3624697

[60] Hinz FI, Aizenberg M, Tushev G, Schuman EM. Protein synthesis-dependent associative long-term memory in larval zebrafish. J Neurosci. 2013;33(39):15382–15387. 24068805 10.1523/JNEUROSCI.0560-13.2013 PMC6618464

[61] Hinz RC, de Polavieja GG. Ontogeny of collective behavior reveals a simple attraction rule. Proc Natl Acad Sci U S A. 2017;114(9):2295–2300. 28193864 10.1073/pnas.1616926114 PMC5338545

[62] Engeszer RE, Barbiano LA, Ryan MJ, Parichy DM. Timing and plasticity of shoaling behaviour in the zebrafish, *Danio rerio*. Anim Behav. 2007;74(5):1269–1275. 18978932 10.1016/j.anbehav.2007.01.032 PMC2211725

[63] Miller N, Gerlai R. From schooling to shoaling: Patterns of collective motion in zebrafish (*Danio rerio*). PLoS One. 2012;7(11):e48865. 23166599 10.1371/journal.pone.0048865 PMC3498229

[64] Nieuwenhuys R . The development and general morphology of the telencephalon of actinopterygian fishes: Synopsis, documentation and commentary. Brain Struct Funct. 2011;215(3-4):141–157. 20976604 10.1007/s00429-010-0285-6 PMC3041917

[65] O’Connell LA, Hofmann HA. Evolution of a vertebrate social decision-making network. Science. 2012;336(6085):1154–1157. 22654056 10.1126/science.1218889

[66] Schmidt R, Strähle U, Scholpp S. Neurogenesis in zebrafish—From embryo to adult. Neural Dev. 2013;8:3. 23433260 10.1186/1749-8104-8-3 PMC3598338

